# Caterpillar-induced plant volatiles attract conspecific adults in nature

**DOI:** 10.1038/srep37555

**Published:** 2016-11-28

**Authors:** Ashraf M. El-Sayed, Alan L. Knight, John A. Byers, Gary J. R. Judd, David M. Suckling

**Affiliations:** 1 The New Zealand Institute for Plant & Food Research Limited, Gerald Street, 7608, Lincoln, New Zealand; 2 USDA-ARS, Agricultural Research Service 5230 Konnowac Pass Rd, Wapato, WA, 98951-9651, USA; 3 Department of Entomology Robert H. Smith Faculty of Agriculture, Food and Environment The Hebrew University of Jerusalem Rehovot, Israel; 4 Agriculture and Agri-Food Canada 4200 Highway 97 Box 5000, Summerland, British Columbia V0H 1Z0, Canada; 5 School of Biological Sciences, University of Auckland Tamaki Campus, Building 733, Auckland, New Zealand

## Abstract

Plants release volatiles in response to caterpillar feeding that attract natural enemies of the herbivores, a tri-trophic interaction which has been considered an indirect plant defence against herbivores. The caterpillar-induced plant volatiles have been reported to repel or attract conspecific adult herbivores. To date however, no volatile signals that either repel or attract conspecific adults under field conditions have been chemically identified. Apple seedlings uniquely released seven compounds including acetic acid, acetic anhydride, benzyl alcohol, benzyl nitrile, indole, 2-phenylethanol, and (*E*)-nerolidol only when infested by larvae of the light brown apple moth, *Epiphyas postvittana*. In field tests in New Zealand, a blend of two of these, benzyl nitrile and acetic acid, attracted a large number of conspecific male and female adult moths. In North America, male and female adults of the tortricid, oblique-banded leafroller, *Choristoneura rosaceana*, were most attracted to a blend of 2-phenylethanol and acetic acid. Both sexes of the eye-spotted bud moth, *Spilonota ocellana*, were highly attracted to a blend of benzyl nitrile and acetic acid. This study provides the first identification of caterpillar-induced plant volatiles that attract conspecific adult herbivores under natural conditions, challenging the expectation of herbivore avoidance of these induced volatiles.

Insect herbivory typically induces a change in the profile of volatile organic compounds (VOCs) released by the affected plants^1^,^2^. Natural enemies of insect herbivores such as parasitoids and predators are attracted to these altered plant odours and as a consequence are better able to locate their herbivorous hosts^3^,^4^,^5^. This tri-trophic relationship is considered an indirect plant defence strategy that recruits natural enemies that incapacitate the herbivores, resulting in higher plant fitness. However, feeding by caterpillars on plants can result in contrasting behaviours by conspecific adult herbivores: (1) In some plant species, volatiles induced by caterpillar feeding repel conspecific adult herbivores. For example, adult moths of several species are repelled by VOCs released by larval feeding including the noctuid cabbage looper, *Trichoplusia ni* (Hübner)^6^, the tobacco budworm, *Heliothis virescens* (Fabricius)^7^, and the fall armyworm, *Spodoptera frugiperda* (J.E. Smith)^8^, as well as the sphingid tobacco hornworm, *Manduca sexta* (Linnaeus)^9^. (2) On the other hand, odours of other plant species infested with caterpillars can attract conspecific adults. For example, mated females of cabbage moth *Mamestra brassicae* (Linnaeus) oriented significantly more to cabbage plants damaged by conspecific larvae compared with undamaged plants^10^. Female Egyptian cottonworm, *Spodoptera littoralis* (Boisduval) moths prefer to oviposit on cotton plants that had been fed on by conspecific larvae compared with non-damaged plants^11^. Similarly, female diamondback moth, *Plutella xylostella* (Linnaeus) prefer to oviposit on conspecific-damaged cabbage plants over undamaged cabbage plants^12^. Tea plants infested with *Ectropis oblique* (Prout) larvae were more attractive to virgin and mated female adults compared to uninfested plants^13^. In none of these field studies were bioactive volatiles chemically identified.

Consistent with the indirect defence theory of tri-tropic interactions, de Moraes *et al*.^7^ proposed that the ability of adult herbivores to detect and avoid oviposition on damaged plants would have several advantages including (a) herbivore offspring avoiding competition for food resources, (b) reducing the probability of encountering natural enemies, and (c) avoiding increased host-plant resistance with lower nutritional content. Regarding the latter advantage, an increase in plant resistance has been linked to the higher production of secondary compounds, mainly monoterpenes, in response to herbivory^14^. Nevertheless, Williams and Myers^15^ found that insect herbivores raised on foliage from trees that were previously infested grew faster and attained heavier pupal weights than those fed foliage from uninfested trees. Perhaps an increase in the emission of monoterpenes from infested plants could cause lower monoterpene concentrations in the foliage resulting in lower resistance to herbivore feeding and benefit attracted herbivores^16^. Therefore, the response of adult herbivores to caterpillar-induced plant volatile compounds remains contentious because of the conflicting behavioural responses of herbivores to induced plant volatiles, as well as the proposed benefits of such behaviours.

Most studies that have isolated and identified herbivore-induced plant volatiles (HIPV) that are behaviourally active in repelling or attracting herbivores have been based solely on laboratory bioassays^1^. To date we know of only two studies that were conducted under field conditions^7^,^17^. However, these field studies did not measure the repellent or attractive behaviours of herbivores to HIPVs, but instead focused on rates of oviposition by herbivores^7^ or the increase in the damage by herbivore feeding^17^. We have not found any reports describing the identification of HIPVs that alter the behaviour of adult herbivores under field conditions. This lack of chemical identification limits our understanding of the evolution of tri-trophic HIPV systems and constrains the delivery of practical monitoring and control methods for pest insects to sustainable agriculture. The identification of behaviourally active HIPVs would also contribute to a better understanding of odorant-based mechanisms involved in host-plant finding and selection.

For our study the initial model involved the light brown apple moth (LBAM), *Epiphyas postvittana*) (Walker) on apples (*Malus domestica* Borkh.) (Rosaceae). This and other leafrollers are economically important pests with an exceptionally wide host range of plant species^18^,^19^. Leafrollers are primarily foliar and quarantine pests of apples (*M. domestica*) and other horticultural crops around the world. Because of the economic importance of apple as an internationally-traded commodity, apple volatiles have been extensively examined, particularly in the context of insect behaviour of codling moth, *Cydia pomonella* (Linnaeus), apple maggot, *Rhagoletis pomonella* (Walsh), and apple sawfly, *Hoplocampa testudinea* (Klug)^20^,^21^,^22^,^23^,^24^,^25^. Seasonal patterns of the release of volatiles from mature apple trees have also been investigated in studies of codling moth and apple fruit attack^26^,^27^. Quantitative and qualitative differences in volatile profiles between apple seedlings infested with LBAM larvae and healthy apple seedlings were recently reported^28^. Experienced parasitoid wasps, *Dolichogenidea tasmanica* (Cameron) chose infested over uninfested apple foliage in a still air bioassay and oriented to infested apple seedlings in a wind tunnel^28^. In that study, however, no specific parasitoid-attractive chemicals were characterized, and the response of adult conspecifics to HIPVs from larval-infested apple seedlings was not investigated. Here, we have focused on the adult herbivore responses to identified HIPVs in the field.

Our initial objective was to investigate whether apple volatiles induced by LBAM larvae in New Zealand aid conspecific adults in locating suitable hosts. We anticipated that our investigation might lead to the identification of a semiochemical for this important pest. Initially, we observed the behaviour of mated female LBAM in response to infested and uninfested apple trees in screened houses. This was followed by chemical characterization of the volatile compounds released uniquely by infested apple seedlings compared with uninfested seedlings. We then tested the unique volatiles of infested apple trees in the field for attraction/repellence of adult male and female moths. These experiments showed robust attraction of LBAM to the tested volatiles. To test how general this phenomenon was we investigated attraction in other economically-important leafrollers in North America, including the obliquebanded leafroller (OBLR), *Choristoneura rosaceana* (Harris) and the eye-spotted bud moth (ESBM), *Spilonota ocellana* (Denis & Schiffermüller).

## Results

### Volatiles emitted by uninfested and larval-infested apple seedlings

Analysis of the headspace of uninfested and infested apple seedlings indicated qualitative differences in odour profiles. We identified a total of 12 compounds in the headspace of uninfested apple seedlings, and 19 compounds in the headspace of infested apple seedlings (Table 1). Acetic acid and acetic anhydride were only detected in SPME samples because there is no co-eluting solvent to overwhelm the GC/MS detector. Otherwise, similar odour profiles were found with regard to quality and ratio of compounds when using SPME or dynamic headspace collection of volatiles. Seven compounds including acetic acid, acetic anhydride, benzyl alcohol, benzyl nitrile, 2-phenylethanol, indole, and (*E*)-nerolidol were present only in volatile emissions from apple seedlings infested with LBAM. In addition to qualitative differences, infestation of apple seedlings with LBAM larvae resulted in alteration of the ratio of the compounds emitted from infested apple seedlings compared with uninfested apple seedlings (Table 1). Acetic anhydride was not tested in field trials because this compound hydrolyses under ambient conditions to produce acetic acid.

Infestation of apple trees with larvae of ESBM and OBLR resulted in both a qualitative and quantitative change in the odour profiles (Table 2). Similar to LBAM infestation, apple trees infested by these two leafroller species released five compounds into the headspace that were not present in headspace from uninfested trees (i.e. benzyl alcohol, benzyl nitrile, 2-phenylethanol, indole, and (*E*)-nerolidol). SPME samples were not performed on headspace samples collected in USA because a GC/MS was not available at USDA Wapato Research Station.

### Response of mated female LBAM to infested and uninfested plants

**Figure 1:**
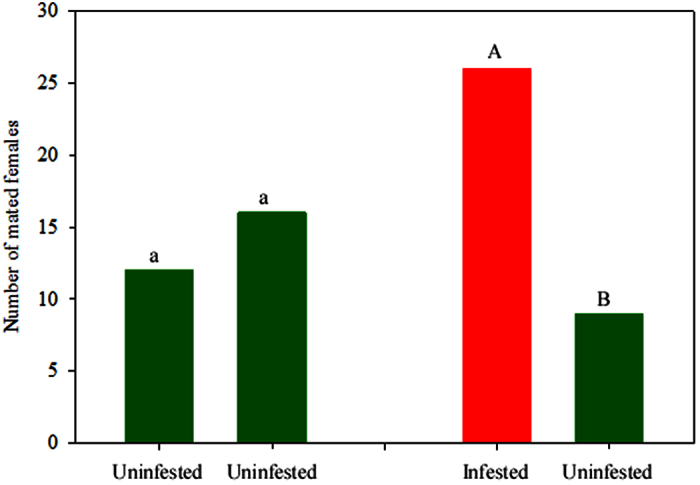
Response of mated LBAM (*Epiphyas postvittana*) (Tortricidae) adult females to infested vs uninfested apple trees in a shade house. In each category, treatments labelled with the same letters are not significantly different (*P* > 0.05). Comparisons were made pairwise and in the first pairwise comparison two “uninfested” were compared to each other.

In a control bioassay, when mated females were presented with two uninfested apple seedlings, similar numbers of mated females were caught on each apple seedling indicating there was no bias in our set-up (Fig. 1, *X*^2^ = 0.98. df = 1, *P* = 0.7). When mated females were presented with uninfested versus infested apple seedlings, significantly more females were caught on sticky panels around the infested seedlings compared to uninfested seedlings (Fig. 1, *X*^2^ = 32.11, df = 1, *P* < 0.001).

### Testing individual HIPV compounds

**Figure 2:**
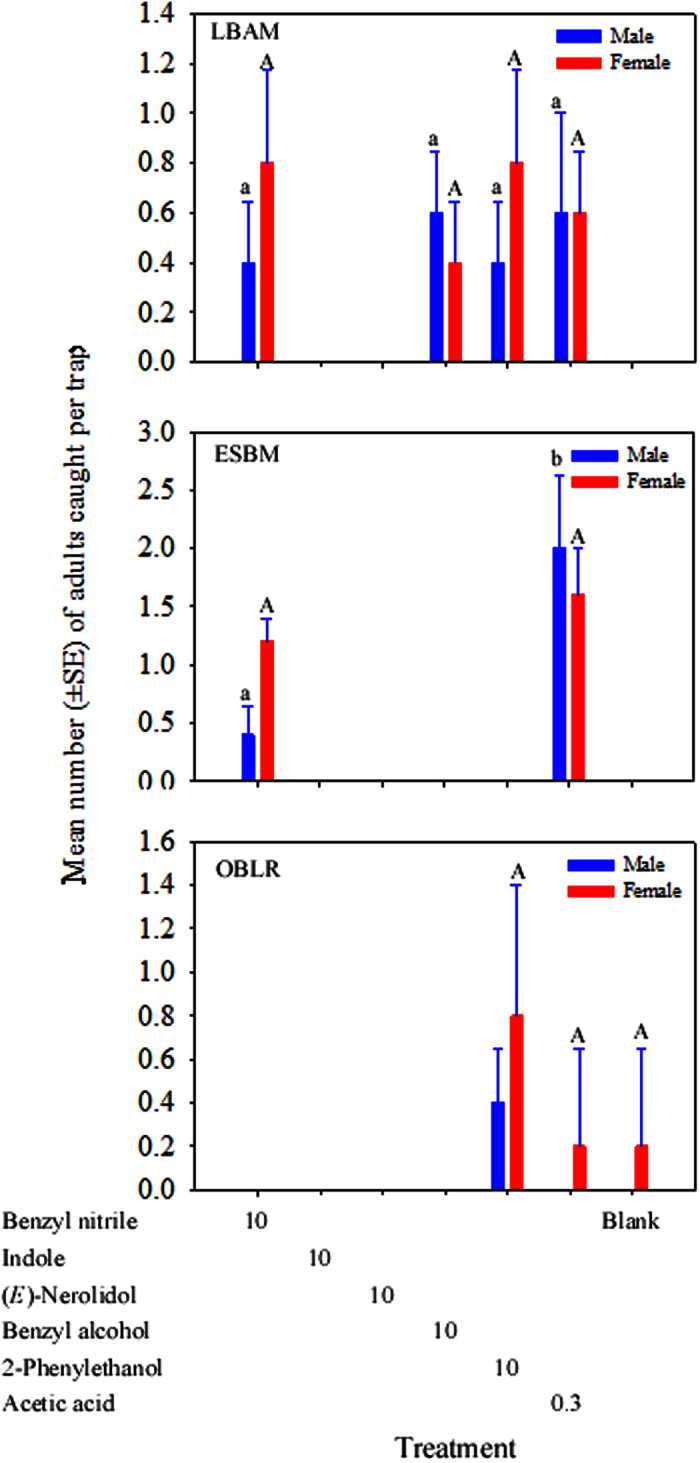
Mean (±SE) of the total number of adult male and female tortricids LBAM (*Epiphyas postvittana*), ESBM (*Spilonota ocellana*), and OBLR (*Choristoneura rosaceana*) caught in traps baited with six specific HIPV compounds in apple orchards. Loading of the first five compounds are in mg, whereas loading of acetic acid is in mL. Treatments labelled with the same case letters are not significantly different (*P* > 0.05). Treatments that caught no moths were not included in the analyses.

Adult LBAM males and females were attracted to each of the four specific HIPV compounds: benzyl nitrile, benzyl alcohol, 2-phenylethanol, and acetic acid, but with no significant differences among compounds (Treatment, *F*_3,16_ = 0.16, *P* = 0.92 for male, *F*_3,16_ = 0.37, *P* = 0.78 for female, Fig. 2). No moths were caught in blank traps. For ESBM, only benzyl nitrile and acetic acid attracted males and females into traps with no significant differences among treatments (*F*_1,8_ = 5.6, *P* = 0.046 for male, and *F*_1,8_ = 0.8, *P* = 0.40 for female, Fig. 2). No ESBM moths were caught in blank traps. For OBLR, only traps baited with 2-phenylethanol attracted males and females, however there were no significant differences among treatments and blank control in the attraction of OBLR females (*F*_2,12_ = 1.16, *P* = 0.35 for female, Fig. 2).

### Testing binary blends of HIPV compounds

**Figure 3:**
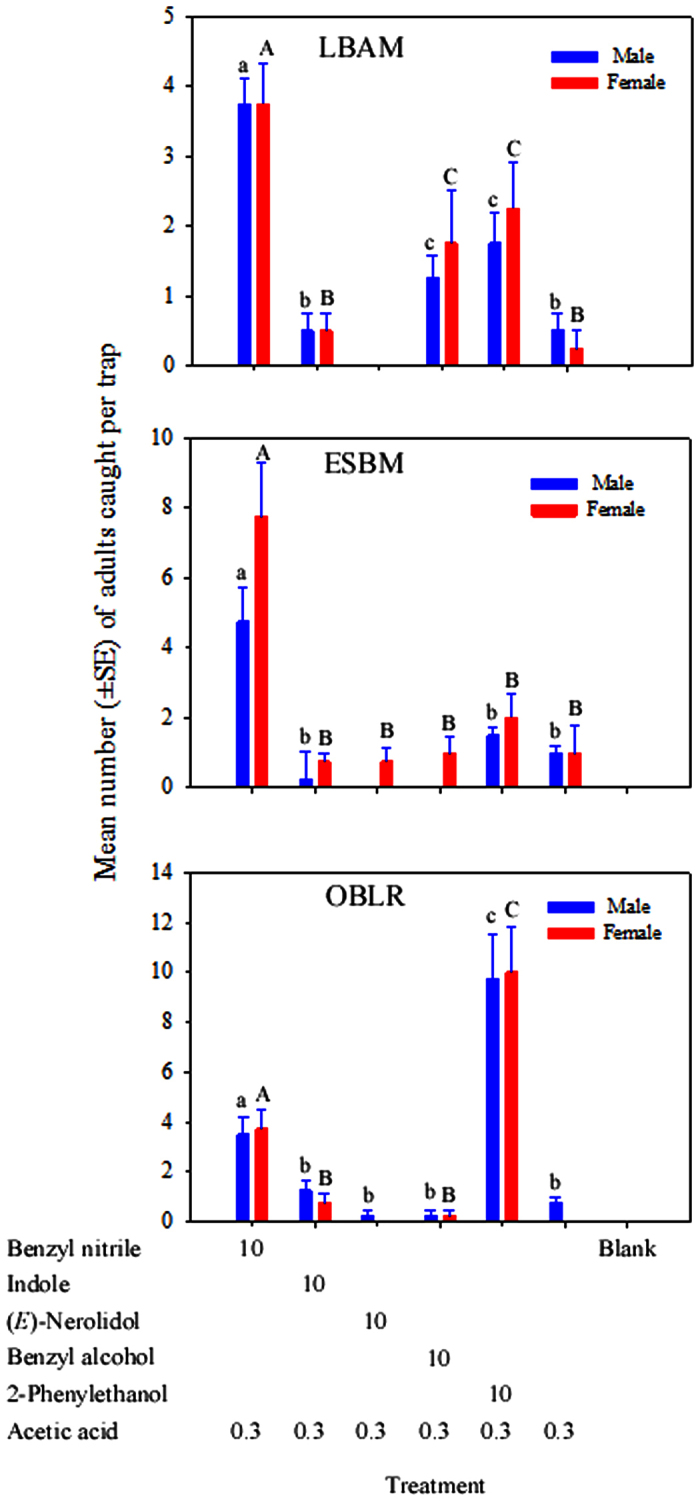
Mean (±SE) of the total number of adult male and female tortricids LBAM (*Epiphyas postvittana*), ESBM (*Spilonota ocellana*), and OBLR (*Choristoneura rosaceana*) caught in traps baited with binary blends containing 10 mg of each HIPV compound +0.3 mL of acetic acid. Treatments labelled with the same case letters are not significantly different (*P* > 0.05). Treatments that caught no moths were not included in the analyses.

The composition of the binary blends altered the number of LBAM, ESBM and OBLR males and females captured (LBAM: Treatment, *F*_4,20_ = 9.2, *P* < 0.001 for male, and *F*_4,20_ = 8.32, *P* = 0.004 for female; ESBM: Treatment, *F*_3,16_ = 10.4, *P* < 0.001 for male, and *F*_5,24_ = 16.29, *P* < 0.001 for female; OBLR: Treatment, *F*_5,24_ = 19.6, *P* < 0.001 for male, and *F*_3,16_ = 15.33, *P* < 0.001 for female). The greatest numbers of adult LBAM and ESBM were captured in traps baited with benzyl nitrile + acetic acid, whereas the greatest numbers of adult OBLR were captured in traps baited with 2-phenylethanol + acetic acid (Fig. 3). For LBAM, the catches in traps baited with the binary blends 2-phenylethanol + acetic acid or benzyl alcohol + acetic acid were greater than from traps baited with acetic acid alone (Fig. 3, *P* < 0.001). The catches in traps baited with indole + acetic acid were not significantly different from traps baited with acetic acid alone (Fig. 3). (E)-nerolidol + acetic acid baited traps caught no moths. For ESBM, the catches with the four binary blends including 2-phenylethanol + acetic acid, benzyl alcohol + acetic acid, indole + acetic acid, or (*E*)-nerolidol + acetic acid were not significantly different from traps baited with acetic acid alone (Fig. 3). For OBLR, the catches in traps baited with the binary blend containing benzyl nitrile + acetic acid were significantly different from traps baited with acetic acid alone (Fig. 3, *P* < 0.001). The catches of adult moths in traps baited with benzyl alcohol + acetic acid, indole + acetic acid, or (*E*)-nerolidol + acetic acid were not significantly different from traps baited with acetic acid alone (Fig. 3, *P* > 0.05).

### Testing quaternary and binary blends of HIPV compounds

**Figure 4:**
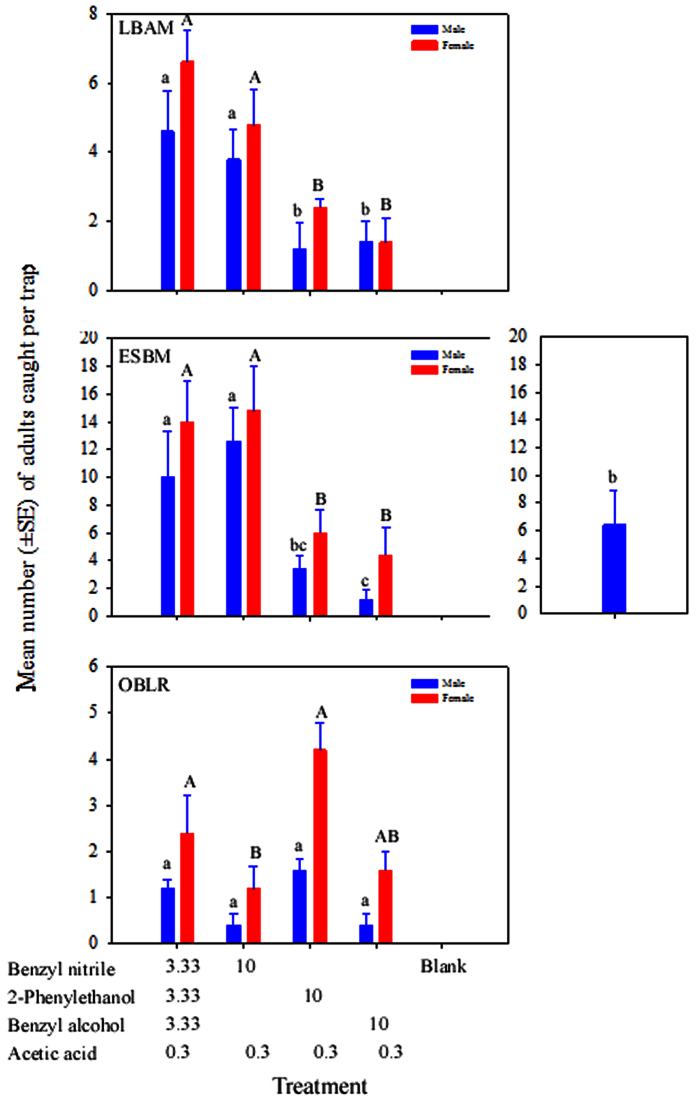
Mean (±SE) of the total number of adult male and female tortricids LBAM (*Epiphyas postvittana*), ESBM (*Spilonota ocellana*), and OBLR (*Choristoneura rosaceana*) caught in traps baited with the quaternary blend and three binary blends of the three HIPV compounds +acetic acid. The box with bar on right represents the male response to sex pheromone. Loading of the first three HIPV compounds are in mg, whereas loading of acetic acid is in mL. Treatments labelled with the same case letters are not significantly different (*P* > 0.05).

The composition of the HIPVs significantly affected the number of adult male and female moths attracted to odour sources (LBAM: Treatment, *F*_3,16_ = 4.3, *P* = 0.021 for male, and *F*_3,16_ = 9.12, *P* < 0.001 for female; ESBM: Treatment, *F*_4,20_ = 4.5, *P* = 0.009 for male, and *F*_3,16_ = 4.4, *P* = 0.019 for female; OBLR: Treatment, *F*_3,16_ = 6.5, *P* = 0.004 for male, and *F*_3,16_ = 5.1, *P* = 0.012 for female). For LBAM and ESBM, the two blends containing benzyl nitrile (binary and quaternary) attracted significantly more adult moths than the two binary blends (2-phenylethanol + acetic acid, benzyl alcohol + acetic acid) (Fig. 4). For OBLR, blends containing 2-phenylethanol (binary and quaternary) attracted significantly more adult moths than the binary blend (benzyl nitrile + acetic acid). (Fig. 4). Both the quaternary blend and binary blend containing benzyl nitrile + acetic acid attracted significantly more ESBM males than traps baited with sex pheromone (*P* < 0.05, Fig. 4).

### Testing synergism between benzyl nitrile and acetic acid

**Figure 5:**
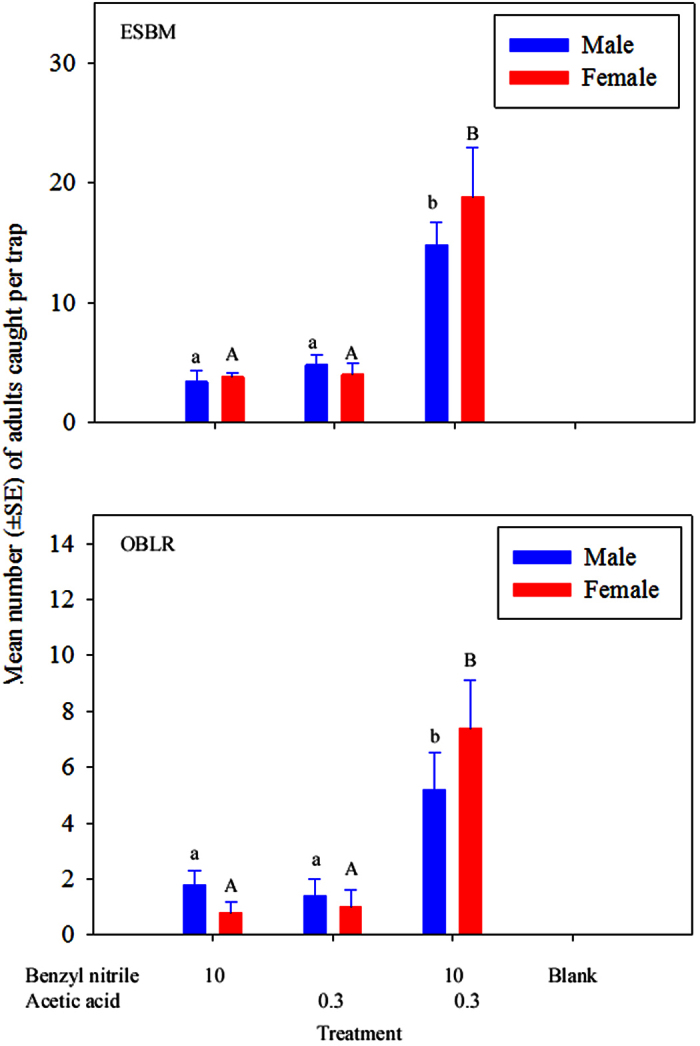
Mean (±SE) of the total number of adult male and female tortricids ESBM (*Spilonota ocellana*) and the OBLR (*Choristoneura rosaceana*), caught in traps baited with, (1) 10 mg benzyl nitrile; (2) 0.3 mL acetic acid; and (3) a binary blend of 10 mg benzyl nitrile + 0.3 mL acetic acid. Treatments labelled with the same case letters are not significantly different (*P* > 0.05).

In the trial investigating the synergism between benzyl nitrile and acetic acid, the composition of the HIPVs significantly affected the number of adult male and female ESBM and OBLR attracted to odour sources (ESBM: Treatment, *F*_2,12_ = 23.4, *P* < 0.001 for male, and *F*_2,12_ = 12.49, *P* = 0.001 for female; OBLR: Treatment, *F*_2,18_ = 6.6, *P* = 0.007 for male, and *F*_2,18_ = 16.7, *P* < 0.001 for female). Benzyl nitrile or acetic acid alone attracted similar numbers of males and females of ESBM or OBLR (Fig. 5). However, benzyl nitrile together with acetic acid resulted in significant increases in the number of males and females caught in both species (*P* < 0.05, Fig. 5).

### Testing various doses of benzyl nitrile plus acetic acid

**Figure 6:**
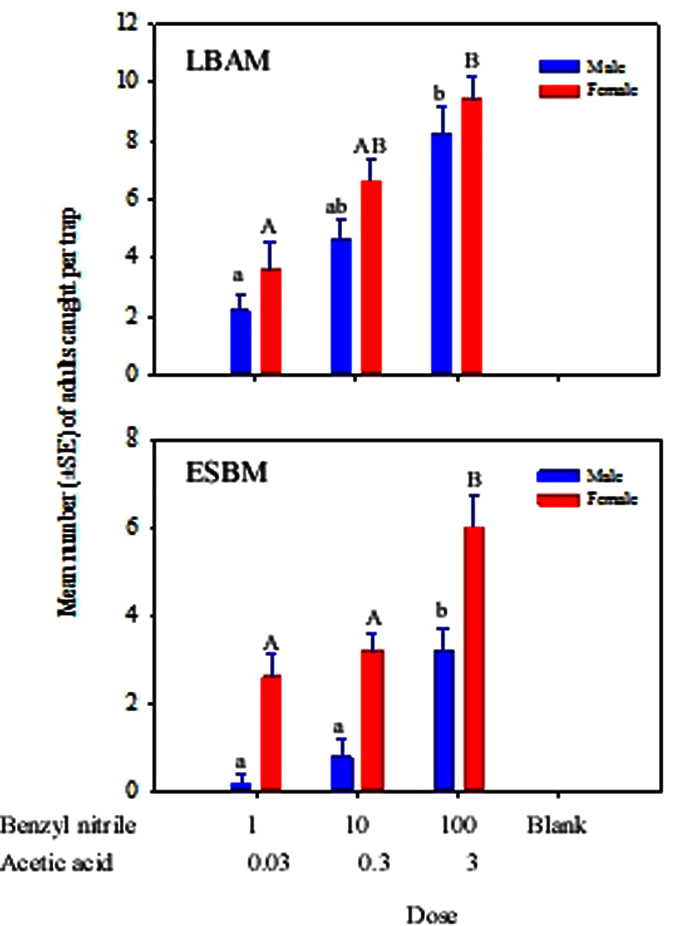
Mean (±SE) of the total number of adult male and female tortricids LBAM, (*Epiphyas postvittana*) and ESBM (*Spilonota ocellana*) caught in traps baited with a binary blend containing three doses of benzyl nitrile + acetic acid (1:0.03, 10:0.3, 100:3 mg:mL). Treatments labelled with the same case letters are not significantly different (*P* > 0.05).

Varying the amount of benzyl nitrile + acetic acid in the binary blend significantly affected the number of males and females of LBAM and ESBM caught (LBAM: Treatment, *F*_2,12_ = 14.8, *P* < 0.001 for male, and *F*_2,12_ = 12.7, *P* = 0.001 for female; ESBM: Treatment, *F*_2,12_ = 18.1, *P* < 0.001 for male, and *F*_2,12_ = 10.9, *P* = 0.002 for female). Increasing the dose of benzyl nitrile + acetic acid from 1 mg + 0.03 mL to 10 mg + 0.3 mL, respectively, did not result in a significant increase in the number of males and females caught (Fig. 6). However, a further increase of benzyl nitrile + acetic acid to 100 mg + 3 mL resulted in a significant increase in the numbers of male and female LBAM and ESBM caught compared to the two lower dosages (Fig. 6).

### Response of other leaf feeding herbivores

In New Zealand, male and female greenheaded leafroller, *Planotortrix octo* (Dugdale), as well as the brownheaded leafroller moths, *Ctenopseustis obliquana* (Walker) and *Ctenopseustis herana* (Felder & Rogenhofer) were caught mainly in traps baited with benzyl nitrile + acetic acid, 2-phenylethanol + acetic acid, and benzyl alcohol + acetic acid (Fig. 1S). In North America, males and females of other species of leafrollers including the three-lined leafroller, *Pandemis limtata* (Robinson), the pandemis leafroller moth, *Pandemis pyrusana* (Kearfott), the European leafroller, *Archips rosanus* (Linnaeus), and the fruit-tree leafroller moth, *Archips argyrospila* (Walker), were caught mainly in traps baited with benzyl nitrile + acetic acid or with 2-phenylethanol + acetic acid (Fig. 3S). Moths in the genus *Pandemis* showed a preference toward 2-phenylethanol + acetic acid, whereas moths of the genus *Archips* showed a preference toward benzyl nitrile + acetic acid (Fig. 3S). Attraction to binary blends of HIPVs was also found in other families of moths. For example, noctuids belonging to genera *Graphania* and *Tmetolophota* were mainly caught in traps baited with benzyl nitrile + acetic acid or with benzyl alcohol + acetic acid (Fig. 2S). Male and female leaf feeding noctuids belonging to genera *Abagrotis*, *Euxoa* and *Agrotis* were caught mainly in traps baited with benzyl alcohol + acetic acid (Fig. 4S). Male and female geometrids, *Anavitrinella spp*. were caught in traps baited with benzyl nitrile + acetic acid (Fig. 4S).

## Discussion

Greater numbers of mated LBAM females were caught at apple seedlings infested with conspecific larvae compared with uninfested seedlings. Qualitative and quantitative differences in the emission of VOCs were observed between apple seedlings infested with LBAM, ESBM and OBLR larvae compared to uninfested seedlings. In this study, we focused on the qualitative rather than quantitative differences between infested and uninfested seedlings. Apple seedlings infested with the three tortricid species emitted seven VOCs including, acetic acid, acetic anhydride, benzyl alcohol, benzyl nitrile, 2-phenylethanol, indole, and (*E*)-nerolidol that are not released by uninfested trees. We report three additional highly volatile low molecular weight compounds (acetic acid, acetic anhydride and 2-phenylethanol) not reported earlier by Suckling *et al*.^28^. All seven compounds were in relatively lower amounts compared to much larger quantities of terpenes (e.g. (*E*,*E*)-α-farnesene, (*Z*,*E*)-α-farnesene, germacrene D, β-caryophyllene, linalool) in the headspace of infested apple trees. Six of the compounds (excluding acetic anhydride that hydrolyses to acetic acid) unique to infested apple seedlings were tested in apple orchards in New Zealand in order to identify a kairomone blend that significantly attracted conspecific LBAM adults of both sexes. Examination of the plant-insect interactions in two North American tortricids, ESBM and OBLR, indicate a similar phenomenon in these species. For LBAM and ESBM, a binary blend of benzyl nitrile and acetic acid was the most attractive to males and females, whereas a binary blend of 2-phenylethanol and acetic acid was the most attractive to OBLR males and females. The catches of ESBM males in traps baited with HIPV compounds were surprisingly higher than the catches in sex pheromone traps, suggesting a potent biological activity for the identified HIPVs, which needs to be confirmed by additional comparisons. Volatiles emitted by damaged leaves of *Fagus sylvatica* L. attracted significantly more European cockchafer, *Melolontha melolontha* L. males than volatiles from intact host plants. However, females were not attracted by any volatiles from damaged or intact leaves of the tested volatile sources^29^. To date, no chemical signals have been identified from infested plants that attract or deter conspecific adult males and females in nature. Most work on herbivore-induced plant volatiles has been undertaken under laboratory conditions. Although some studies have been conducted in the field, these have measured rates of herbivore oviposition or feeding damage on plants in the presence of HIPV compounds^7^,^17^. The previous lack of identified field-active chemical cues that directly attract or repel herbivores could be due to several reasons, such as incomplete chemical identification of odours from infested plants (in our case acetic acid was critical for eliciting appropriate behavioural response) or testing suboptimal blend combinations and dosages in field trials. Our study presents the first identification of biologically active VOCs from infested plants that attract adult herbivores in nature.

The two benzene derivatives, benzyl nitrile and 2-phenylethanol have been widely reported as herbivore-induced plant volatile compounds in many plant species (e.g.^30^,^31^,^32^,^33^,^34^,^35^,^36^,^37^). In black poplar, both benzyl nitrile and 2-phenylethanol are biosynthesized from the precursor L-phenylalanine by CYP79/CYP71 enzymes with phenylacetaldoxime as an intermediate^38^. Benzyl nitrile elicits strong electrophysiological responses in several parasitic wasps^39^,^40^, while acting as an attractant for hymenopteran parasitoids of herbivores in the field^36^. Acetic acid was present only in infested plants and has been reported as an induced plant volatile compound produced by apple trees under attack of spider mites, *Amblyseius andersoni* (Chant) and *Amblyseius californicus* (McGregor)^41^. The production of acetic acid in infested plants could be due to higher bacterial abundance associated with larval feeding compared to uninfested plants. Acetic acid is usually produced by aerobic bacteria through oxidation of ethanol or by anaerobic bacteria directly converting sugars to acetic acid during fermentation. Acetogenic bacteria can also produce acetic acid from methanol or carbon monoxide. Therefore, the bioactive blends identified in this study could be purely HIPV compounds or a combination of HIPV compounds and fermentation products associated with larval feeding.

Benzyl nitrile, 2-phenylethanol and acetic acid showed less biological activity when each was tested alone in our bioassay. In addition to being HIPVs, or in the case of acetic acid as a possible bacterial fermentation product, these three compounds are common in nature as floral volatiles or fermentation products, not necessarily associated with herbivory^42^,^43^. The general abundance of these three compounds would reduce the distinctiveness of such chemical signals, which might explain the lower activity of these compounds when tested alone. Certainly the binary blends of acetic acid and either benzyl nitrile or 2-phenylethanol resulted in a significant increase in the number of conspecific adults caught. The addition of acetic acid to benzyl nitrile and 2-phenylethanol would enhance the uniqueness and increase the apparency of the chemical signal against background chemical noise. Acetic acid has been reported as an insect kairomone for many insect species, mainly when combined with other compound(s)^42^. For example, isobutanol is attractive to various social wasps of genus *Vespula spp*. and the addition of acetic acid to this compound results in 16-fold and 20-fold increases in the capture of German yellowjacket, *Vespula germanica* (Fabricius) and Western yellowjacket, *Vespula pensylvanica* (Saussure), respectively^44^. Social wasps are carnivores and prey mostly on caterpillars; the identification of acetic acid as a caterpillar-induced compound in our study indicates that the compound might act as attractant for social wasps, hence the synergistic effect of acetic acid on the response of social wasps to isobutanol. Similarly, the addition of acetic acid to ethyl (*E*,*Z*)-2,4-decadienoate (pear ester) or pear ester and (*E*)-4,8-dimethyl-1,3,7-nonatriene results in a significance increase in the number of male and female codling moths attracted to pear ester^45^,^46^. In contrast to social wasps, feeding is not critical for adult codling moth^47^, and therefore acetic acid probably acts as a long range chemical cue to locate a suitable oviposition site.

Adult herbivores display a range of responses to plants infested with conspecific larvae depending on the species of herbivore and plant. In some cases, adult herbivores are attracted to plants infested with larvae^10^,^11^,^12^,^13^, whereas others are repelled by infested plants^6^,^7^,^8^,^9^. The contrasting responses of adult herbivores to plants infested with conspecific larvae could be due to differences in herbivore biology, where some species prefer to lay their eggs in masses, e.g. fallweb worm *Hyphantria cunea* (Drury), so that larvae aggregate in a confined space for protection and overcoming host resistance, while other species lay their eggs individually to avoid competition, e.g. codling moth, *C. pomonella*. There may well be fitness advantages to being deterred by such compounds, including avoiding competition as well as predation and parasitism from natural enemies attracted by the HIPVs. However, the potency of the binary blends in attracting conspecific adults was a surprise and raises an important question. What are the advantages for conspecific adult herbivores that are attracted to infested plants? The majority (>85%) of LBAM females caught in traps baited with HIPVs were mated (El-Sayed unpublished data) suggesting that most of those females were seeking oviposition sites to lay their eggs. Infested plants might be more favourable oviposition sites because plant resistance may be much lower and survival higher than on healthy uninfested plants^15^,^16^. It is not clear why males were also attracted to these compounds. Their attraction could be due either to (a) an artefact in which females in the traps released sex pheromone before dying (although unlikely as they were mated) or (b) natural selection in which there is a higher probability of encountering a female attracted to the infested plants compared to uninfested plants^48^.

During the course of this study, large numbers of heterospecific adult males and females of thirteen different herbivore species in several families were caught in traps baited with HIPV compounds (Supplementary Material). It is important to note that all these herbivores are leaf feeders. There was a degree of variation among the response of the heterospecific adult herbivore to HIPV compounds. In general, leafrollers and geometrid species showed preferences towards blends of benzyl nitrile + acetic acid and 2-phenylethanol + acetic acid, whereas noctuid species showed preferences towards benzyl alcohol + acetic acid. Therefore, further research is required to determine whether these herbivores are (a) exploiting host susceptibility due to infestation with heterospecific caterpillars, (b) the HIPV compounds are similar to the compounds produced by the infestation of conspecific caterpillars, or (c) the oviposition location cues are shared across many moth species. Female biased receptor neurons have been found on the antennae of female, silkworm, *Bombyx mori* (Linnaeus) that respond to benzene-derived compounds including 2-phenylethanol^49^. The positive response of three conspecific and thirteen heterospecfic herbivores to HIPV compounds indicate that this phenomena is widespread at least among leaf-feeding moths.

The extensive literature published on plant-mediated multi-trophic interactions between plants, herbivores and natural enemies suggests a paradigm in which plants release a bouquet of HIPV compounds upon feeding by herbivores. These HIPV compounds have been implicated as foraging cues for natural enemies to locate herbivore hosts, while deterring conspecific herbivores. Such a system would result in enhancing the plant’s defence against herbivore attack. The apple-mediated multi-trophic interaction between plant-leafroller herbivores investigated in our study deviates from this scenario, with the chemical cues released from infested plants attracting the leafroller herbivores. The level of attraction of the three leafrollers to caterpillar-induced plant volatiles provides the strongest evidence to date demonstrating a positive attraction rather than a deterrent effect in the plant-herbivore-natural enemy interaction model. Our results further reinforce this attractive effect with a number of species in three families of Lepidoptera that also exhibit this phenomenon. These results may suggest a reevaluation is needed of the general deterrent model of responses of adult herbivores to HIPV compounds. Further work is required to determine whether females oviposit or males mate at the infested plants as well as rates of parasitoid/predator attraction and leafroller population reduction. These studies will help to better understand the counterbalancing adaptive factors affecting herbivore infestations. Furthermore, our identification of potent kairomones that attract both male and female leafrollers can greatly improve the applied use of semiochemicals for control of these important pests distributed worldwide. Formulations containing both sex pheromones and kairomones could be tested for mass trapping or lure and kill tactics against these pests^50^,^51^.

## Methods

### Plants and insects

A colony of LBAM was established and maintained at Mt. Albert Research Centre (Auckland). Egg batches of *E. postvittana* were allowed to emerge in the laboratory. Apple seedlings (var. Royal Gala) were infested with ca. 30 larvae (mix of first and second instar) per seedling and left for 24 h in the laboratory to enable larvae to settle. Upon making contact with the adaxial leaf surface, larvae orientated to a position on the abaxial surface where they webbed up along the midrib and began to feed on epidermal tissue. Visible signs of larval feeding were evident after 24 h. Infested and uninfested apple seedlings (30–50 cm high) were used to provide volatile odour profiles by air filtration and in the greenhouse trials. Pupae from the colony were separated by sex and adult males were allowed to emerge in isolation from the females. Emerging adults were used to produce mated females for the shade house bioassay. One female and three males were confined in a plastic container for one night and monitored with a time lapse video system. Females and males were considered mated when they were observed in copula for more than one minute. Adults were kept at 22 ± 2 °C, 18 L:6D, and provided with water until testing at 2–3 days post eclosion.

For eye-spotted bud moth, before apple bud break in spring of 2014, several hundred 30-cm branch sections were pruned from mixed blocks of organic Gala and Ambrosia apple orchards located near Cawston in the Similkameen Valley of British Columbia (49°13′N, −119°58′W). Pruned branches consisting mainly of fruit-spur wood were transported to the laboratory and placed in a controlled-environment chamber at 19 °C under a 13:11 h L:D photoperiod provided by Daylight fluorescent tubes. On each collection date, branches were placed in a plastic basin (35 cm × 35 cm × 16 cm), covered with polyester organza and held in place with an elastic band that prevented larva from escaping but permitted air circulation. Every 24 h, branches were removed from their basin and tapped sharply to dislodge active larvae onto a white cloth. Upon emergence from their overwintered hibernaculae, larvae were moved to 2 oz plastic cups (Solo Cup Company, Lake Forest, IL) maintained at outside ambient light and temperature conditions. Larvae were supplied with fresh mixed-variety apple leaves collected from the surrounding organic orchards for food and nesting material. Apple leaves were sprayed with a 2% bleach solution and rinsed to remove potential contaminants before introduction. Leaves were replenished every 2–3 days until pupation. Upon adult eclosion mixed-sex moths were moved to mass-mating chambers. *S. ocellana* adults were mass mated in plastic cages (640 ml) lined with plastic bags and sealed with a mesh nylon screen. Females deposited eggs onto plastic which lined the mating chambers. Plastic liners were replaced daily to ensure fresh eggs were always available. Egg sheets with 1-day-old eggs were couriered to Yakima, WA, USA.

Obliquebanded leafroller. *C. rosaceana* were obtained from a laboratory colony at Washington State University, Wenatchee, WA that was established in 1990 from larvae collected from apple orchards in Mattawa, WA. This colony has been reared continuously since their collection on a pinto bean diet following the method of Shorey & Hale^52^ under constant conditions of temperature (23 ± 2 °C), relative humidity (RH, 70%), photoperiod (16:8, L:D), and without exposure to insecticides. Neonate ESBM and 4th instar OBLR were transferred with a brush to new shoots on 2-year-old, potted ‘Fuji’ apple trees at the USDA Laboratory in Wapato, WA. Three to five larvae were transferred to each actively-growing shoot on several trees.

### Chemicals

Chemical purity of the standards used to identify the compounds in infested apple seedling headspace and used in the field experiments were as follows: Glacial acetic acid (99%), benzyl alcohol (99%), (*E*)-nerolidol (85%), benzyl nitrile (99%), 2-phenylethanol (99%), and indole (99%). Glacial acetic acid was stored under ambient temperature while all other compounds were stored at −20 °C until used. All compounds were purchased from Sigma Aldrich (MO, USA).

### Air Entrainment of volatiles emitted by apple seedlings infested with LBAM larvae

Infested and uninfested apple seedlings (ca. 30 cm high, cv. Royal Gala) were enclosed for 48 h in individual 14 id × 50 high cm glass chambers (ten replicates). Each chamber inlet was fitted with an air filter consisting of 1.5 mm granular activated charcoal (Merck, Darmstadt, Germany), contained in a 14 × 3 cm diam. two-piece glass cylinder, which could be readily opened. Filtered air (1 L min^−1^) was drawn into the sampling chamber with a CF-MP1 sampling pump (Brey, Memmingen, Germany) and upward and over the leaves of the seedling. A volatile entrainment filter, comprising a glass tube containing 100 mg of Tenax-GR 35/60 mesh (Grace, Deerfield, IL) between glass wool plugs, was attached to the sampling chamber outlet. For qualitative and quantitative analyses, the Tenax entrainment filters were washed with 1 mL of n-hexane (BDH Laboratory Supplies, Poole, U.K.), containing 500 ng of dimethyl salicylate as the internal standard. This standard was chosen because it was not seen in the extracts yet and is similar in structure and retention times to some of the volatiles. The resulting eluate was stored at −80 °C until analysis by gas chromatography/mass spectrometry (GC/MS). Sampling filters were reused after cleaning by flushing with nitrogen (50 mL min^−1^) in an oven heated at 200 °C for 16 h. After each sampling occasion, all glassware were disassembled and glass and charcoal were heated at 140 °C in an oven for 16 h before reuse. The total leaf area (cm^2^) was determined for apple seedlings using a LI-3000 portable area meter (Lambda Instruments Corp., Lincoln, NE).

### Sampling of volatiles emitted by apple seedlings infested with LBAM larvae by SPME

Infested and uninfested apple seedlings (ca. 30 cm high, cv. Royal Gala) were enclosed for 24 h in individual 14 × 50 cm glass chambers (five replicates). No air flow was allowed into these chambers and both inlet and outlet were closed. SPME (solid phase microextraction) analysis used a 50/30-μm divinylbenzene/carboxen/polydimethylsiloxane fiber (Supelco Inc., Bellefonte, PA) for headspace sampling around the foliage. The SPME fiber was inserted from the top of the chamber and was exposed to each sample for 5 min at ambient temperature (23–27 °C), after which each sample was injected into a GC/MS injection unit. SPME fibers were conditioned prior to sample collection by injecting the fiber in the GC inlet for 1 hour at 220 °C.

### Air entrainment of volatiles emitted by apple seedlings infested with ESBM, and OBLR larvae

Volatile collections from infested apple trees (cv. Red Jonaprince) with either ESBM, or OBLR larvae and uninfested apple trees were conducted in Yakima, USA using a dynamic headspace collection method, where air containing the odor was passed over and absorbed by a sorbent filter, followed by solvent extraction. Intact tree branches with either apple leaves infested with leafroller larvae or uninfested leaves were enclosed in a polyester oven bag (Glad NZ^®^, 35 cm × 50 cm). A charcoal–filtered air stream was pulled over the enclosed leaves at 0.5 L/min, and the headspace volatiles were collected for 24 h on an adsorbent filter containing 50 mg of Tenax-GR 35/60 (Alltech Associates Inc.) in a 60 mm long × 6 mm diameter glass tube. For collection of control samples, a charcoal–filtered air stream was pulled through an empty oven bag in the same greenhouse. Samples were sealed and shipped in dry ice to Plant and Food Research (PFR) facility for GC/MS analysis. At the PFR lab, the Tenax filters were extracted with 0.5 ml of n-hexane (AnalaR BDH, Laboratory Supplies, Poole, UK). A sub-sample of 100 μl was reduced to 10 μl at ambient temperature under a stream of argon and 1 μl of the concentrated extract was injected in the GC/MS. Six volatile collection samples from infested and uninfested leaves and six control samples were taken.

### Analysis of air-entrainment samples by Gas Chromatography/Mass Spectrometry (GC/MS)

The concentrated extracts of the air-entrainment samples were analyzed using GC/MS (Varian 3800 GC coupled to a Varian 2200 MS). Helium was used as the carrier gas (1 mL min^−1^), and injections were splitless for 0.6 min. Transfer line and ion trap temperatures were 250 and 180 °C, respectively. The GC injector temperature was set at 220 °C, and the oven ramp was 40 °C for 2 min, 4 °C min^−1^ to 240 °C, hold for 10 min, and then 15 °C min^−1^ to 260 °C, using a VF-5 MS capillary column (30 m × 0.25 mm inner diameter × 0.25 μm film; and a polar 30 m × 0.25 mm i.d. × 0.5 μm, VF23-MS capillary column; Varian, Inc., Walnut Creek, CA). A 1 μL aliquot was injected after first concentrating 100 μL of each sample to ca. 10 μL with a gentle stream of argon. The spectra were recorded at an ionization voltage of 70 eV over a mass range mass-to-charge (*m/z*) of 20 to 499. Kovats retention indexes (KI) were calculated for the compounds^43^ (Table 1). Structural assignments of the compounds were made by comparing their mass spectra with NIST 2005 MS library, as well as by comparison to Kovats retention indices published in the literature^43^. Identification of volatiles was confirmed by comparison to authentic samples.

### Response of mated female LBAM to infested and uninfested plants

This experiment was conducted from 16 January to 20 February 2012 in a screened house (4 × 6 m) to determine the response of mated female LBAM to infested and uninfested apple trees. Two apple seedlings (infested and uninfested) ~1 m high were placed in each side of the screened house. Infested apple seedlings were inoculated with 60 second instar LBAM larvae 72 h before use. The two apple seedlings were separated by about 3 m and were covered by net to prevent oviposition on uninfested apple trees. Mated females were released in the centre of the screened house. Initially we tested uninfested vs uninfested seedlings to determine if there was any bias in our setup, then we tested uninfested vs. infested seedlings. Four transparent sticky cylinders (8-cm diameter x 21-cm high) coated with sticky polymers (Tanglefoot Company, Grand Rapids, MI) made of plastic sheet vertically positioned about 50-cm above ground on a 60-cm wooden pole around each tree. Five replicates each of 75 mated females were released every five days over a five week period. Sticky panels were checked each week and new trees were used every day and tree position was rotated.

### Field experimental protocol

All field experiments targeting LBAM were conducted in a ‘Red Delicious’ apple orchard (43°38′55.39″S, 172°27′17.57″E) in Canterbury, the South Island, New Zealand. All field experiments targeting ESBM and OBLR were conducted in a mixed variety organic apple orchard ((49°13′N, −119°58′W) in Cawston, British Columbia, Canada unless stated otherwise. Red delta traps made of plastic corflute with an adhesive-coated base^53^ were used in LBAM trials while large white delta traps (Pherocon VI, Trécé Inc, Adair, OK) were used for ESBM and OBLR trials. Traps baited with different treatment blends of HIPV compounds in five replicates were assigned in five rows, each containing treatments tested in a randomized block design. Traps were positioned 1.7 m above the ground in each trap tree, and were spaced 20 m apart in each row. The polyethylene sachets were placed in the center of the sticky base in the LBAM trials while it was attached to the side of the traps using Velcro tape in ESBM and OBLR trials. In trials lasting more than a week, sticky bases were checked, replaced and counted weekly during the experimental period.

### Testing individual HIPV specific compounds

This experiment was conducted from 28 November to 12 December 2014 for LBAM and 3–10 June 2015 for ESBM and OBLR to investigate the biological activity of the individual HIPV compounds. Lures of benzyl alcohol, benzyl nitrile, indole, 2-phenyethanol, (*E*)-nerolidol, and 0.3 mL acetic acid were made by dispensing 10 mg of each compound on a OCB regular cellulose acetate filter (6 mm diameter × 15 mm, OCB, Chicago, USA) inside a permeable polyethylene bag (100 μm wall thickness, 20 mm × 20 mm, Brampton, ON, Canada) that was then heat-sealed. A trap baited with a blank lure was used as negative control.

### Testing binary blends of HIPV compounds

A second field experiment was conducted between 15 December 2014 to 13 January 2015 for LBAM and from 12 to 23 June 2015 for ESBM and OBLR to investigate binary blends of the most attractive compounds obtained in the first trial. The loadings of the five HIPV blends were prepared similar to the first experiment as follows: 1) 10 mg benzyl alcohol + 0.3 mL acetic acid; 2) 10 mg benzyl nitrile and 0.3 mL acetic acid; 3) 10 mg indole + 0.3 mL acetic acid; 4) 10 mg 2-phenyethanol + 0.3 mL acetic acid; and 5) 10 mg (*E*)-nerolidol and 0.3 mL acetic acid. A trap baited with 0.3 mL of acetic acid alone and a blank lure were used as controls.

### Testing quaternary and binary blends of HIPV compounds

This experiment was conducted between 15–28 January 2015 for LBAM and from 26 June to 6 July 2105 for ESBM and OBLR. We investigated the synergistic effect of combining all active compounds in trial 2 in one quaternary blend vs three binary blends. The four HIPV volatile blends were: (1) 3.33 mg benzyl nitrile, 3.33 mg 2-phenylethanol, 3.33 mg benzyl alcohol + 0.3 mL acetic acid; (2) 10 mg benzyl nitrile + 0.3 mL acetic acid; (3) 10 mg 2-phenylethanol + 0.3 mL acetic acid; and (4) 10 mg benzyl alcohol + 0.3 mL acetic acid. A trap with a blank lure was used as negative control, while traps baited with the sex pheromone of ESBM AlphaScents) were used as positive control.

### Testing synergism between benzyl nitrile plus acetic acid

This experiment was conducted from 21 June to 2 July 2015 only for ESBM and OBLR in a mixed varieties apple orchard in Summerland, British Columbia, Canada (49°34′50.02″N, 119°38′16.57″W). The relative attractiveness of the following treatments was investigated: (1) 10 mg benzyl nitrile alone; (2) 0.3 mL acetic acid alone; and (3) 10 mg benzyl nitrile + 0.3 mL acetic acid. A trap with a blank lure was used as negative control.

### Testing various doses of benzyl nitrile plus acetic acid

This experiment was conducted between 30 January to 16 February 2014 for LBAM and from 15–28 July 2105 for ESBM and OBLR. The relative attractiveness of three doses of binary blends containing (1 + 0.03; 10 + 0.3; and 100 mg + 3 mL) of benzyl nitrile + acetic acid was investigated. Traps baited with a blank lure were used as a negative control.

### Data analysis

The variance of mean captures obtained with each compound or each blend of compounds was stabilized using √ (x + 1) of counts for tests of significance of treatments using ANOVA. Significantly different treatment means were identified using Tukey HSD test was used to identify significantly different means using JMP software Version 9.0.1 (SAS Institute Inc., Cary, USA).

## Additional Information

**How to cite this article**: El-Sayed, A. M. *et al*. Caterpillar-induced plant volatiles attract conspecific adults in nature. *Sci. Rep*. **6**, 37555; doi: 10.1038/srep37555 (2016).

**Publisher’s note:** Springer Nature remains neutral with regard to jurisdictional claims in published maps and institutional affiliations.

## Supplementary Material

Supplementary Information

## Figures and Tables

**Table 1:** Relative amounts (% ±SE) and release rate (pg h^−1^ cm^−2^ of leaf area, ±SE) of the compounds identified in the headspace of apple seedlings with and without infestation by LBAM (*E. postvittana*) larvae using two volatile collection methods (SPME and dynamic headspace collection).

Compound	RI^1^	SPME headspace Relative amounts (%)^2^		Dynamic headspace (pg h^−1^ cm^−2^)	
		Uninfested	Infested	Uninfested	Infested
Acetic acid		nd^3^	0.73 ± 0.2	—	—
Acetic anhydride		nd	1.11 ± 0.1	—	—
(*Z*3)-Hexenyl acetate	1006	4.30 ± 1.21	5.72 ± 1.0	4.68 ± 1.26	10.52 ± 3.35
Benzyl alcohol	1037	nd	0.83 ± 0.2	nd	2.00 ± 0.90
2-Phenylethanol	1047	nd	0.35 ± 0.1	nd	1.77 ± 0.86
(*E*)-beta-Ocimene	1101	11.62 ± 4.63	9.62 ± 2.2	0.83 ± 0.26	9.63 ± 3.23
Linalool	1107	3.74 ± 1.31	2.24 ± 1.3	2.81 ± 0.76	5.50 ± 3.68
(*E*)-4,8-Dimethyl-1,3,7-nonatriene	1116	4.21 ± 1.35	4.03 ± 1.2	3.68 ± 0.49	6.71 ± 4.02
Benzyl nitrile	1146	nd	0.76 ± 0.2	nd	1.37 ± 0.62
Methyl salicylate	1195	3.91 ± 0.81	2.12 ± 0.5	1.59 ± 0.42	3.63 ± 1.28
Indole	1288	nd	0.75 ± 0.2	nd	1.37 ± 0.63
β-Caryophyllene	1423	4.23 ± 2.13	1.82 ± 0.7	0.99 ± 0.20	2.56 ± 0.81
Germacrene D	1486	3.02 ± 1.11	2.03 ± 0.4	1.08 ± 0.41	5.42 ± 2.51
(*Z,E*)-α-Farnesene	1493	7.53 ± 3.04	6.11 ± 0.9	6.14 ± 2.12	15.42 ± 5.37
(*E,E*)-α-Farnesene	1507	57.31 ± 7.32	62.63 ± 4.1	29.89 ± 5.93	66.37 ± 21.36
(*E*)-Nerolidol	1564	nd	0.92 ± 0.22	nd	2.53 ± 1.04
(*Z*3)-Hexenyl benzoate	1575	0.32 ± 0.12	0.62 ± 0.28	0.22 ± 0.10	0.78 ± 0.45

^1^Kovats Retention Index (VF5-MS capillary column).

^2^Percentage of total volatiles produced are given as means area of the GC peaks followed by the standard errors (n = 6).

^3^not detected.

**Table 2:** Relative amounts (% ± SE) of the compounds identified in the headspace of apple trees with and without infestation by ESBM (*S. ocellana*), and OBLR (*C. rosaceana*).

Compound	Dynamic headspace Relative amounts (%)^2^				
	RI^1^	*S. ocellana*		*C. rosaceana*	
		Uninfested	Infested	Uninfested	Infested
(*Z*3)-Hexenyl acetate	1006	7.44 ± 1.26	9.12 ± 3.10	6.40 ± 1.47	3.15 ± 1.22
Benzyl alcohol	1037	nd^3^	1.22 ± 0.42	nd	0.89 ± 0.26
2-Phenylethanol	1047	nd	0.93 ± 0.31	nd	1.36 ± 0.46
(*E*)-beta-Ocimene	1101	2.47 ± 0.64	1.93 ± 0.31	1.93 ± 0.28	2.32 ± 0.82
Linalool	1107	5.77 ± 0.94	3.92 ± 0.96	3.70 ± 1.34	7.42 ± 1.89
(*E*)-4,8-Dimethyl-1,3,7-nonatriene	1116	3.32 ± 0.69	1.92 ± 0.21	8.43 ± 4.2	2.65 ± 0.44
Benzyl nitrile	1146	nd	0.89 ± 0.40	nd	0.53 ± 0.32
Methyl salicylate	1195	3.74 ± 1.78	1.92 ± 0.14	5.91 ± 1.33	2.68 ± 0.95
Indole	1288	nd	0.93 ± 0.37	nd	0.79 ± 0.52
β-Caryophyllene	1423	2.14 ± 0.74	0.75 ± 0.50	2.25 ± 0.95	2.81.6 ± 0.46
Germacrene D	1486	5.29 ± 1.46	0.82 ± 0.19	6.10 ± 1.39	4.9 ± 0.77
(*Z,E*)-α-Farnesene	1493	9.88 ± 2.87	4.78 ± 1.77	4.65 ± 1.39	4.78 ± 1.92
(*E,E*)-α-Farnesene	1507	59.14 ± 5.82	69.04 ± 6.22	60.01 ± 6.71	64.51 ± 3.38
(*E*)-Nerolidol	1564	nd	1.30 ± 0.44	nd	0.56 ± 0.19
(*Z*3)-Hexenyl benzoate	1575	0.81 ± 0.26	1.25 ± 0.34	0.45 ± 0.26	0.67 ± 0.31

^1^Kovats Retention Index (VF5-MS capillary column).

^2^Percentage of total volatiles produced are given as means area of the GC peaks followed by the standard errors (n = 5).

^3^not detected.
