# Supplementary material for: Caterpillar-induced plant volatiles attract conspecific adults in nature
Source: Sci Rep. 2016 Nov 28;6:37555. doi: 10.1038/srep37555 (PMC5124949; doi:10.1038/srep37555)
Supplement: Supplementary Information [file srep37555-s1.pdf]

Supplementary materials

Caterpillar-induced plant volatiles attract conspecific adults in nature

Ashraf M. El-Sayed[1*], Alan L. Knight[2], John A. Byers[3], Gary J.R. Judd[4] and David M. Suckling[1,5]

[1]The New Zealand Institute for Plant & Food Research Limited,
Gerald Street, 7608,
Lincoln, New Zealand

[2]USDA-ARS, Agricultural Research Service
5230 Konnowac Pass Rd, Wapato,
WA, 98951-9651, USA

[3]Department of Entomology
Robert H. Smith Faculty of Agriculture, Food and Environment
The Hebrew University of Jerusalem
Rehovot, Israel

[4]Agriculture and Agri-Food Canada
4200 Highway 97 Box 5000,
Summerland, British Columbia V0H 1Z0, Canada

[5]School of Biological Sciences, University of Auckland
Tamaki Campus, Building 733,
Auckland, New Zealand

[*]Author for correspondence email: *ashraf.el-sayed@plantandfood.co.nz*

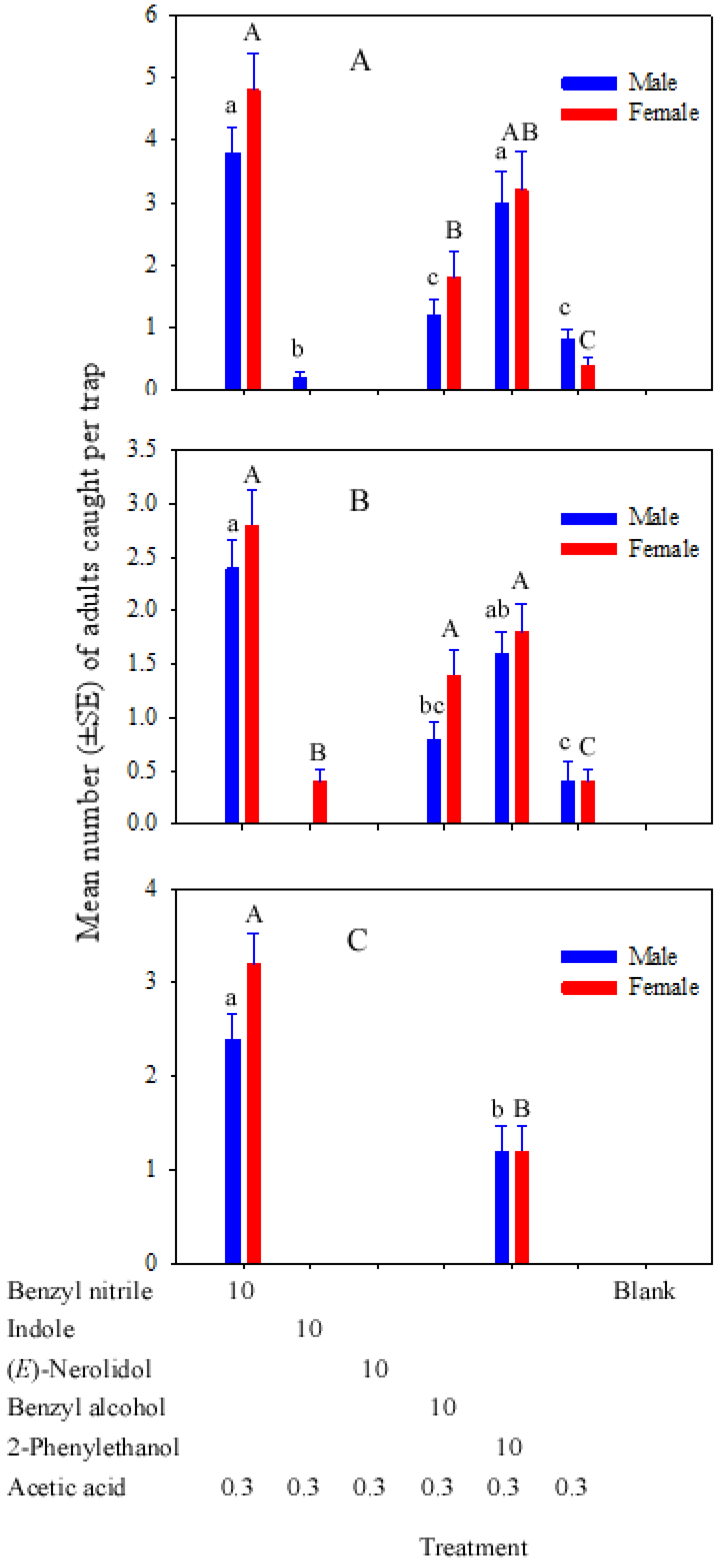

Fig. 1S. Mean (±SE) of the total number of males and females of *Planotortrix octo* (A), *Ctenopseustis obliquana* (B), and *Ctenopseustis herana* (C) caught in traps baited with binary blends containing 10 mg of each HIPV compound + 0.3 mL of acetic acid. Treatments

labelled with the same case letters are not significantly different (P > 0.05). Treatments that caught no moths were not included in the analyses.

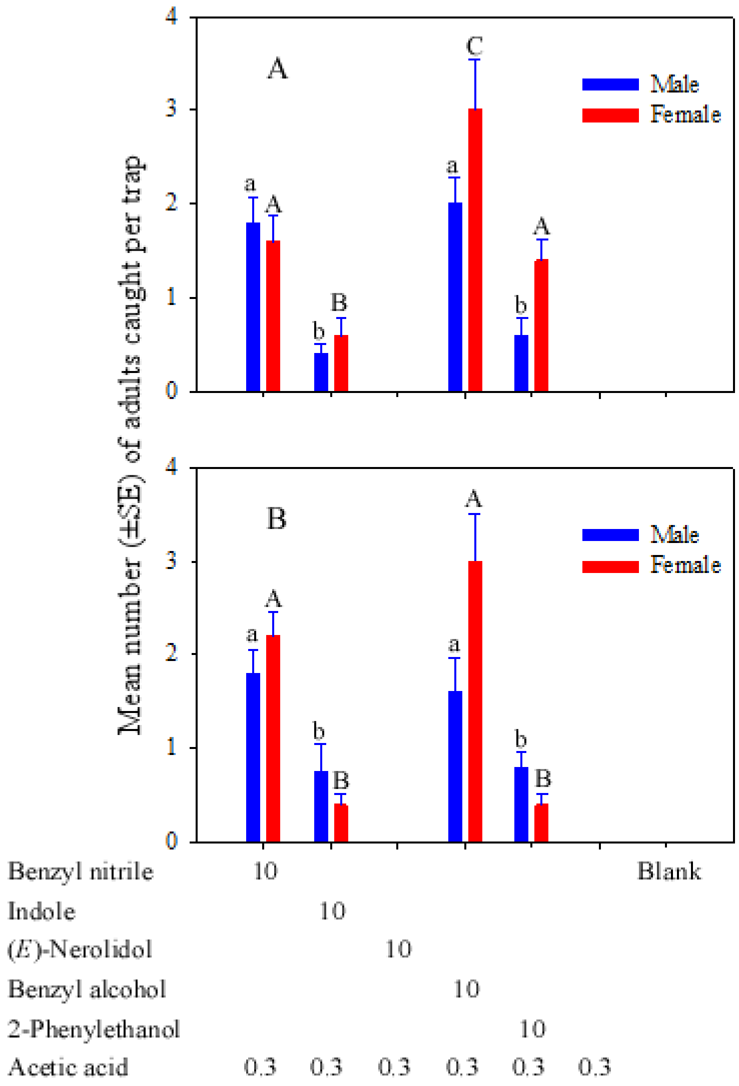

Fig. 2S. . Mean (±SE) of the total number of males and females of *Graphania spp*. (A), and *Tmetolophota spp*. (B) caught in traps baited with binary blends containing 10 mg of each HIPV compound + 0.3 mL of acetic acid. Treatments labelled with the same case letters are not significantly different (P > 0.05). Treatments that caught no moths were not included in the analyses.

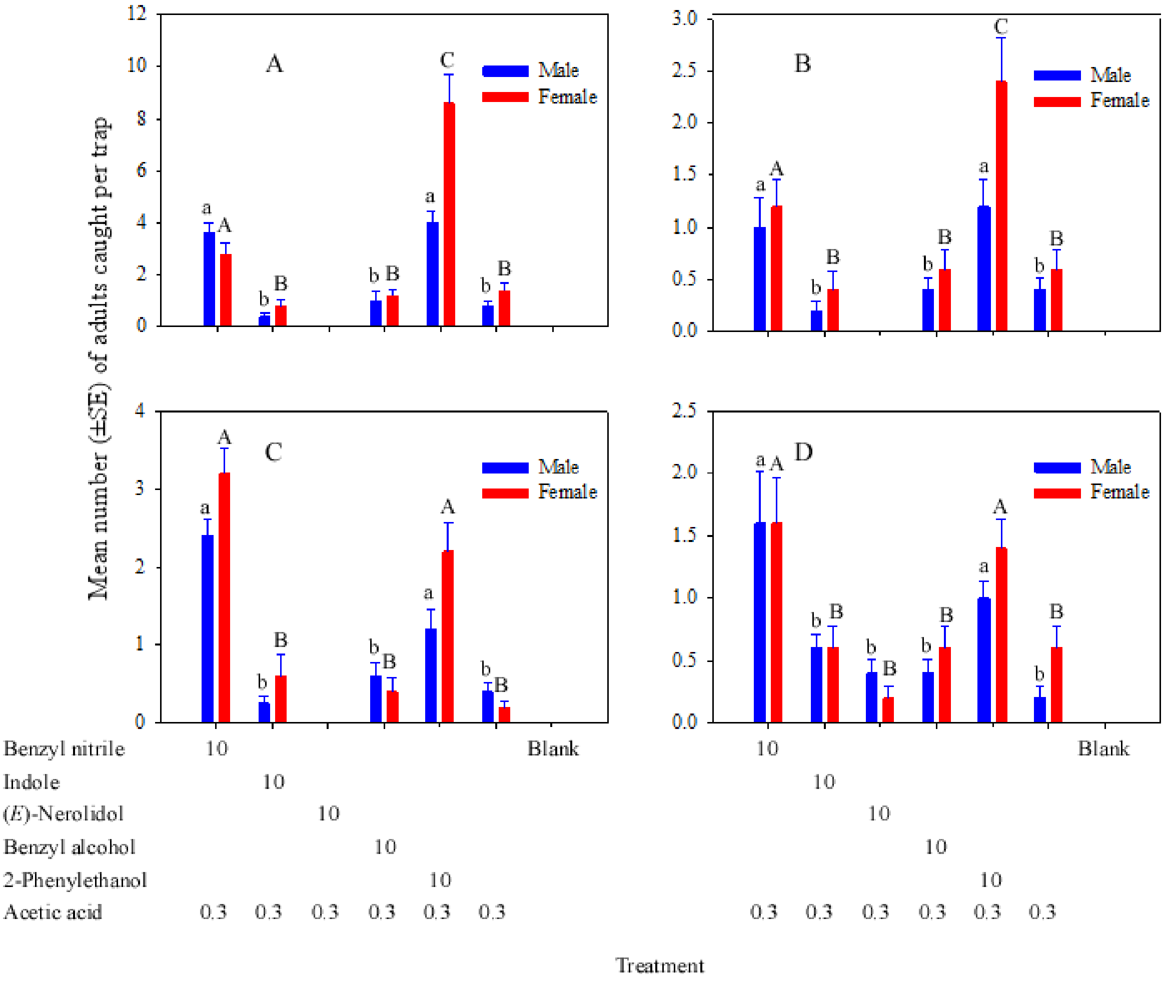

Fig. 3S. . Mean (±SE) of the total number of males and females of the pandemis leafroller moth, *Pandemis pyrusana* (A), the three-lined leafroller, *Pandemis limtata* (B), the European leafroller, *Archips rosanus* (C) and the fruit-tree leafroller moth, *Archips argyrospila* (D) caught in traps baited with binary blends containing 10 mg of each HIPV compound + 0.3 mL of acetic acid. Treatments labelled with the same case letters are not significantly different (P > 0.05). Treatments that caught no moths were not included in the analyses.

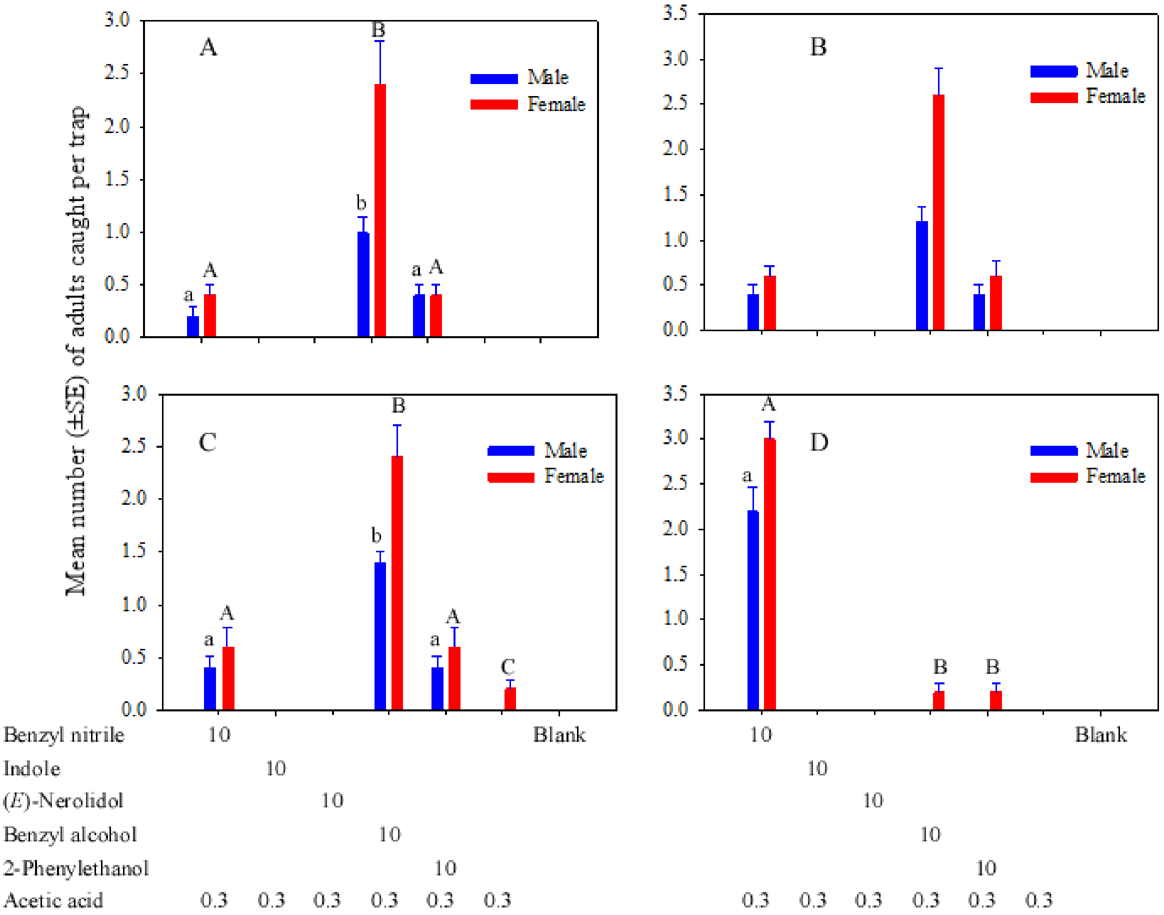

Fig. 4S. Mean (±SE) of the total number of males and females of *Abagrotis spp.* (A), *Euxoa spp.* (B), *Agrotis spp.* (C) and *Anavitrinella spp.* (D) caught in traps baited with binary blends containing 10 mg of each HIPV compound + 0.3 mL of acetic acid. Treatments labelled with the same case letters are not significantly different (P > 0.05). Treatments that caught no moths were not included in the analyses.

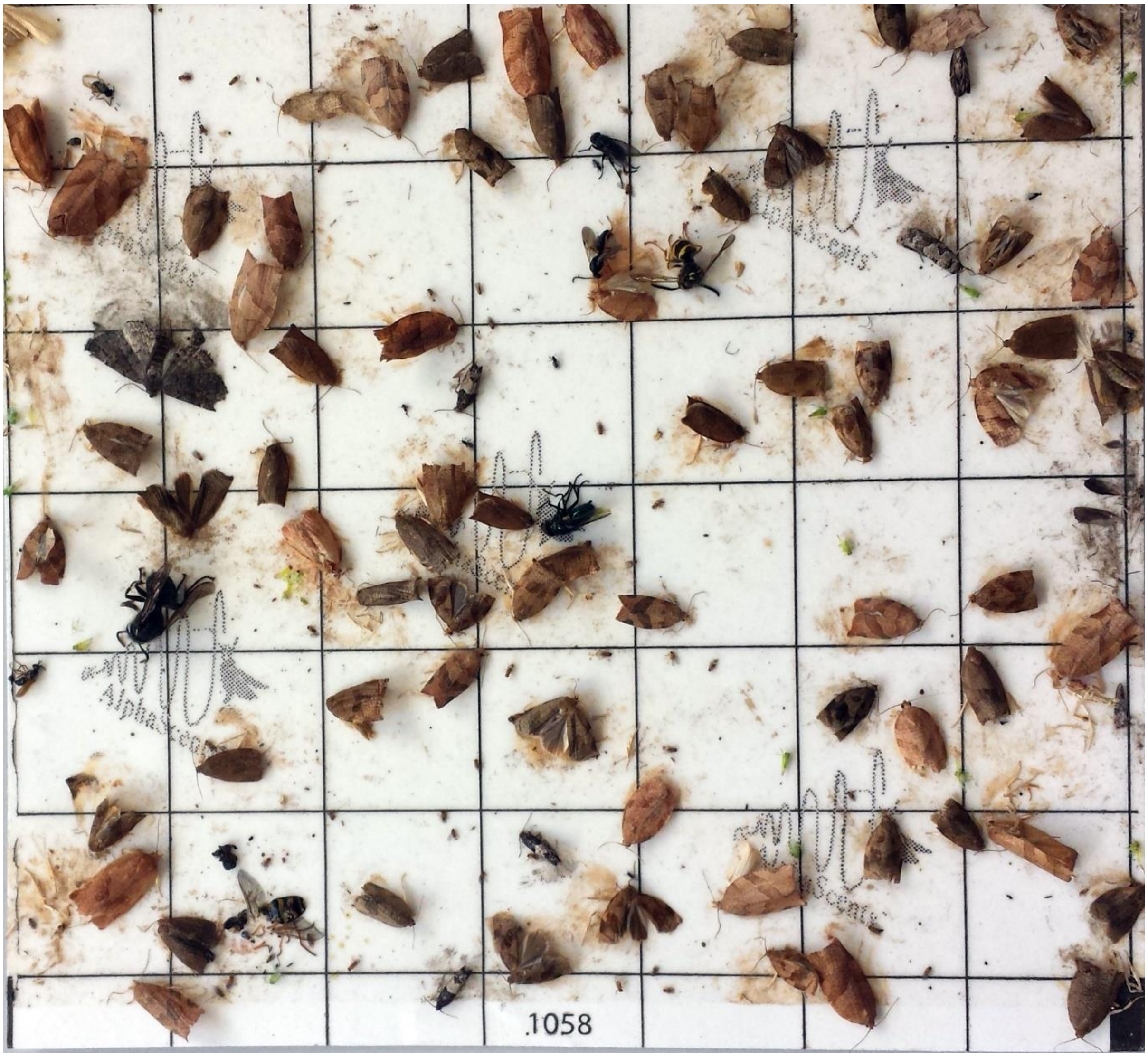

Fig. 5S. Unprecedented wide range of leafrollers females were caught in traps baited with HIPV compounds
